# Does training increase the use of more emotionally laden words by nurses when talking with cancer patients? A randomised study

**DOI:** 10.1038/sj.bjc.6600412

**Published:** 2002-07-15

**Authors:** D Razavi, N Delvaux, S Marchal, J-F Durieux, C Farvacques, L Dubus, R Hogenraad

**Affiliations:** CP191, Université Libre de Bruxelles, Faculté des Sciences Psychologiques et Pédagogiques, 50 av, Franklin Roosevelt, Brussels, 1050 Belgium; Hôpital Universitaire Erasme, Service de Psychologie, Brussels, Belgium; C.A.M., Groupe de Recherche et de Formation, Brussels, Belgium; Université Catholique de Louvain, Faculté de Psychologie et des Sciences de l'Education, Louvain-La-Neuve, Belgium

**Keywords:** empathy, training, communication skills, cancer, computer-supported content analysis

## Abstract

The emotional content of health care professionals–cancer patient communication is often considered as poor and has to be improved by an enhancement of health care professionals empathy. One hundred and fifteen oncology nurses participating in a communication skills training workshop were assessed at three different periods. Nurses randomly allocated to a control group arm (waiting list) were assessed a first time and then 3 and 6 months later. Nurses allocated to the training group were assessed before training workshop, just after and 3 months later. Each nurse completed a 20-min clinical and simulated interview. Each interview was analysed by three content analysis systems: two computer**-**supported content analysis of emotional words, the Harvard Third Psychosocial Dictionary and the Martindale Regressive Imagery Dictionary and an observer rating system of utterances emotional depth level, the Cancer Research Campaign Workshop Evaluation Manual. The results show that in clinical interviews there is an increased use of emotional words by health care professionals right after having been trained (*P*=0.056): training group subjects use 4.3 (std: 3.7) emotional words per 1000 used before training workshop, and 7.0 (std: 5.8) right after training workshop and 5.9 (std: 4.3) 3 months later compared to control group subjects which use 4.5 (std: 4.8) emotional words at the first assessment point, 4.3 (std: 4.1) at the second and 4.4 (std: 3.3) at the third. The same trend is noticeable for emotional words used by health care professionals in simulated interviews (*P*=0.000). The emotional words registry used by health care professionals however remains stable over time in clinical interviews (*P*=0.141) and is enlarged in simulated interviews (*P*=0.041). This increased use of emotional words by trained health care professionals facilitates cancer patient emotion words expressions compared to untrained health care professionals especially 3 months after training (*P*=0.005). This study shows that health care professionals empathy may be improved by communication skills training workshop and that this improvement facilitates cancer patients emotions expression.

*British Journal of Cancer* (2002) **87**, 1–7. doi:10.1038/sj.bjc.6600412 www.bjcancer.com

© 2002 Cancer Research UK

## 

The need for improving ‘health-care professionals (HCP)– cancer patients (CP)’ communication has been frequently emphasized in oncology (Maguire *et al*, 1996; Parle *et al*, 1997). It is assumed that the quality of this communication is linked to CP quality of life and recovery. It has been recently shown for example that an enhanced compassion videotape may decrease viewers – breast cancer survivors –anxiety (Fogarty *et al*, 1999). It is also assumed that the numerous professional stress factors met with in oncology can affect the quality of HCP communication in their clinical practice.

It has been often hypothesized that empathy and the facilitation of CP expression of emotional concerns/distress is one of the factors which may improve the quality of HCP communication. HCP usually block CP expression of emotional concerns/distress and are often unable to clarify CP emotional concerns/distress not only because they fear to acknowledge emotions but also because they feel uncomfortable speaking about emotions when they are openly expressed (Razavi *et al*, 2000). This discomfort is also related to HCP difficulty to deal with their own emotions and to their attempt to suppress their own emotions.

There is a need to better understand the expression of emotional concerns/distress in the context of HCP–CP communication. Moreover, there is a need to help HCP to cope with his own and others emotions, to facilitate acknowledgment of emotions, and to clarify emotional concerns/distress. HCP training is therefore certainly on the agenda of psychiatrist and psychologist working in clinical settings devoted to cancer care.

During the last decade, several research programmes have been designed to assess the effectiveness of training workshops (TW) aiming at the improvement of HCP communication skills (CS). These TW are designed for HCP who wish to improve their CS (Maguire and Faulkner, 1988; Razavi *et al*, 1988, 1991, 1993; Razavi and Delvaux, 1997; Fallowfield *et al*, 1998). In particular, more knowledge is needed on the impact of HCP improved communication skills on CP emotional concerns/distress expression.

Available tools for this purpose have been often constructed to assess significant levels of emotional concerns/distress (mainly anxiety and depression symptoms). Low and moderate levels of emotional concerns/distress are more difficult to assess. Counting emotional words may be a way to assess the emotional tone of a text (Osgood *et al*, 1957; Heise, 1965; Gottschalk and Bechtel, 1982). Emotional content may be also present in larger semantic unit such as utterances (Anderson and McMaster, 1982; Whissell and Dewson, 1986; Whissell *et al*, 1986; Martindale, 1987; Hogenraad and Bestgen, 1989; Bestgen, 1994). The comparison of the performance of content analysis systems and their respective benefit to assess the enhancement of HCP empathy by TW is still a matter of debate.

There are numerous skills related to professional empathy. Some skills do not necessarily need the use of emotional words : for example taking patient perspective and feeling patient emotions in a given context. Some others need the ability to use emotional words: patient emotions recognitions, replications, discriminations and supportive responses. The ability of using emotional words is thus an important part of professional empathy. In theory this ability may facilitate patient expression of emotions. This study was designed to assess TW impact on HCP and CP use of emotional words and on the emotional depth of their utterances. It was hypothesized that HCP would be more able to use emotional words after training. It was also hypothesized that this increased ability would be associated with an increased use of emotional words by CP.

## SUBJECTS AND METHODS

### Training aims and content

In the present study, training workshop (TW) were designed to teach HCP more appropriate communication skills (CS). Registration to TW was open to HCP who wished to develop their knowledge about the psycho-social issues that are related to cancer disease diagnosis and progression. At the end of these TW, participants were expected to feel more comfortable in interacting with patients, and particularly in clarifying their concerns. They were also expected to acquire a basic understanding of the main psychological and psychiatric dimensions related to cancer diagnosis and progression. The aim of the programme was thus to improve health care professionals CS in general and empathy in particular.

TW included a total of 3 weeks training (each week including five consecutive days): 1 week for each of the three consecutive months. The programme included theoretical information (30 h) on one hand and on the other hand, experiential exchanges (case presentations) and role-playing exercises (75 h). A total of 40 role playing exercises were scheduled; each subject participated in four exercises. The programme is described in a detailed manual (available by writing to the authors). Aims, contents and techniques were standardised in order to allow module replication.

In order to make a structured progression between training sessions, the following themes were successively approached: basic communication components in oncology; risk factors related to cancer (genetic, biological, environmental and psychological); psychosocial dimensions associated to cancer diseases sites, stages and treatments; psychological dimensions associated to pain and other symptoms (nausea, vomiting, anorexia..); psychiatric complications and their treatment (pharmacological management of the psychiatric disorders); psychological adjustment mechanisms and concept of death; psychotherapeutic interventions; interventions for specific problems like self image, sexuality; familial reactions to cancer and grief; stress and its consequences for health care professionals; ethical issues related to informed consent, therapeutic determination, euthanasia. During the TW, only two theoretical courses (about pain, other symptoms control and psychiatric disorders) were presented by a pain specialist and a psychiatrist.

The trainer was an experienced psychologist trained in psycho-oncology and skilled in group training. To standardise TW, he was the only trainer of all the 12 groups (6 experimental groups and 6 control groups). The trainer has been specifically trained for the present project : information about the research design (methodology and tools), training group observations, participation to the elaboration of the manual describing the TW. This preliminary training was followed by regular supervision of the trainer.

### Study design

The research protocol has been approved by the Ethical Committee of Jules Bordet Institute, Cancer Center of the University of Brussels. A longitudinal and randomised design was used in which nurse candidates for a TW were allocated to a 105-h training group or to a 6-month waiting list group. The candidates were assigned randomly to a Training Group (TG) or to a Control Group (CG). TG and CG were assessed three times: at baseline (T1), 3 months after T1 (T2) and 6 months after T1 (T3). Nurses allocated to the training group (TG) were thus assessed before TW (T1), just after TW (T2) (3 months after T1) and 3 months later (T3). Nurses allocated to the control group (CG) were trained after having completed all their assessments. The timepoints for assessments are thus similar for both groups.

### Subjects

The protocol was presented to the nursing managers in 88 hospitals. These institutions were chosen on basis of the ‘Annuaire Statistique des Hôpitaux’ established by the Belgian Ministry of the Public Health and Environment according to the following criteria: having treatment facilities for cancer patients, capacity of 60 beds at least, location in the French-speaking part of Belgium or the Brussels area. Standardised information sessions (duration: 90 min; content: information and discussion about the project) were organized for nurses working with cancer patients. The aim of the standardised information sessions was to invite nurses to participate in a controlled study assessing the efficacy of a 105-h TW. Interested health care professionals were registered, after receiving the agreement of the nursing manager, and assigned to the Training Group (TG, *n*=57) or the Control Group (CG, *n*=58). The effective inclusion in the project was confirmed after an individual interview between the candidates and the study co-ordinator. The subjects were active nurses experienced in caring for cancer patients for at least 6 months and wishing to participate in a psychological training group. Professional stability was required during the training period. They understood that the training would be offered and that they had to remain available during a 6-month period for the assessment procedures.

### Assessments

All the subjects were invited to a three-point assessment procedure. The trainer was never involved in the assessment procedure. TG subjects were assessed 1 week before training (T1), 1 week after the end of training (T2) and 3 months later (T3). For the CG subjects, assessments were scheduled at the same time intervals (3 and 6 months after baseline assessment). Each assessment included for the TG and CG subjects the recording of a role playing with an actor (a 20-min simulated interview) and with a cancer patient (a 20-min clinical interview). A same actor was performing at each assessment timepoint for all nurses.

The 20-min videotaped role-playings and audiotaped clinical interviews were retranscribed by 10 secretaries. Moreover, interviewed cancer patients were invited to fulfill the EORTC QLQ-C30 questionnaire (Aaronson *et al*, 1993). This questionnaire was used in the present study to describe not only the emotional functioning but also the physical functioning and the symptoms profile of patients who accepted to participate in recorded interviews. EORTC QLQ-C30 has thus been used to control possible functioning profile differences between patients included in the study at each assessment point (especially the emotional profile which is reflected by the emotional functioning score).

The Cancer Research Campaign Workshop Evaluation Manual (CRCWEM) (Booth and Maguire, 1991), which was translated and adapted into French (Razavi *et al*, 1993), was used to assess the emotional depth of utterances. The raters were blind for the TG or CG status of the subjects and for the assessment time (T1, T2 or T3). The CRCWEM provides a rating of form, function, content and psychological depth of each utterance of an interview. The CRCWEM contains four levels of emotional depth (0,1,2 and 3): level 0 refers to a neutral emotional content of the utterance, level 1 refers to an implicit painful emotional experience, level 2 refers to an explicit painful emotional experience and level 3 refers to an explicit and very painful emotional experience. Content analysis of the emotional depth level (EDL) of utterances with the CRCWEM provides two emotional scores for each interview. The first score is the total number of utterances referring to emotional levels 1,2, or 3 (implicit and explicit emotions). The second score is the total number of emotional levels 2 and 3 (explicit emotions only).

The Harvard Third Psychosociological Dictionary (HPSD) (3513 words) is composed of 83 tags or content categories into which selected words can be tallied if found in the text. Stone *et al* (1966) stated the structure of these tags is two-fold. First-order tags are in reference to the explicit, denotative meanings of words and are further broken into objects (nouns) that are sociological in definition, processes (verbs) reflecting psychological activities, and qualifiers. Second-order tags identify pervasive qualities of the text and refer to both denotative and connotative meanings. Whereas the words in first-order tags are mutually exclusive, words in the second-order tags are not independent of first-order tags or each other. Thus, the first-order tags reflect the structure of the language used, while second-order tags describe qualitative aspects of the document. The ‘Dictionnaire Psycho-sociologique de Harvard’ (3223 words) (Hogenraad *et al*, 1995), is the French version of the HPSD. In this study only the content category ‘distress’ was used (210 words), which is a first-order tag.

Martindale (1975) Regressive Imagery Dictionary (MRID) contains 2900 words assigned to 43 tags reducible to 10 summary categories: ‘emotions’ is one of these summary categories. The dictionary was designed to tap psychoanalytic processes and content in language. The ‘Dictionnaire d'Imagerie Régressive’ (DIRE) (Hogenraad and Orianne, 1986) is the French version of the Regressive Imagery Dictionary (Martindale, 1975, 1979, 1990) and includes 2484 words. In this study the following subcategories of the summary ‘emotion’ category were used: ‘aggression’ (211 words), ‘anxiety’ (46 words), ‘sadness’ (55 words), and ‘positive affect’ (73 words).

PROTocol ANalyzer (PROTAN) (Hogenraad *et al*, 1995) is a programme for Content Analysis that counts the number of words that correspond to the words in the categories defined by the dictionaries. Segmentation units considered in this study are HCP or patient utterances, and each interview produces scores for HCP and patients utterances. Computer-supported analysis with HPSD and MRID provides, using the formula (f/N) × 1000 (where f is the number of tagged words and N the total number of words included in HCP or patients utterances of a given interview), a frequency or density of tagged words per 1000 words. The ‘frequency’ refers to the total number of emotional words and the ‘density’ refers to the total number of different emotional words.

The EORTC QLQ-C30 is a quality of life self-assessment for cancer patients. This questionnaire includes 30 items and has been validated in a cancer patient population (Aaronson *et al*, 1993). This questionnaire has been developed on the basis of a multidimensional definition of quality of life. It includes five subscales related to functioning (physical, emotional, role, cognitive), a global assessment subscale quality of life of nine isolated items assessing symptoms. High subscale scores represent a high level of symptoms; high functioning subscale scores and quality of life global assessment represent a high level of functioning and a positive quality of life.

### Statistical Analysis

Statistics were computed through the SPSS-PC software. To be evaluable subjects had to participate to at least 12 days of the (15 days) 3 weeks training programme. Counting total numbers of words was used to provide descriptive statistics. Partial correlations were used, first to correlate dictionary scores of CP and HCP segments, and secondly to correlate EDL-CRCWEM scores and dictionary scores. Multivariate analyses of variance (MANOVA) were used to compare mean scores in clinical and simulated interviews.

## RESULTS

### Descriptive data

One hundred and twenty-five nurses from 33 hospitals were potentially eligible for the study. Following the inclusion visit, nine subjects were not included in the study for the following reasons : fear of role playing technic (*n*=1), fear of audio- and videotaped interviews (*n*=2), work load (*n*=4), lack of personal motivation (*n*=1), other ongoing training (*n*=1). One hundred and sixteen nurses were included in the study. One subject randomised in the TG participated for only 1 training week. One hundred and fifteen nurses were thus evaluable for the purpose of the study. Fifty-seven subjects were randomised in the TG and 58 in the CG. Baseline socio-demographic characteristics (age, sex, marital status, education) as well as socio-professional characteristics (type of service, professional status, experience with cancer patients) were similar for both randomised groups.

The socio-demographic characteristics of cancer patients (age, sex, educational level and setting of the interview) who participated at the different assessment points (at T1, *n*=114; at T2, *n*=111; at T3, *n*=110) to the recorded interview with a health care professional were also similar in the TG and the CG. There is no group by time effect for the different levels of functioning (physical, role, emotional, cognitive, social) assessed by the EORTC QLQ-C30 (Manova *F* value: 1.81; *P*= 0.07). There is moreover no group by time effect for emotional functioning scores (Manova *F* value: 0.50; *P*=0.602). There is also no group by time effect regarding the physical symptoms items scores of the EORTC QLQ-C30 (fatigue, nausea and vomiting, pain, dyspnea, sleeping problems, constipation, diarrhoea, appetite problems) and the financial impact score (Manova *F* value: 0.64; *P*=0.85).

### Group by time effects

In clinical interviews (CI), trained HCP use less neutral utterances (level 0) than untrained HCP especially 3 months after training (T3) (group by time effect Manova *F* value: 3.00, *P*=0.055). No significant group by time effect is noticed in simulated interviews (SI).

In simulated interviews (SI) and clinical interviews (CI), HCP utterances include approximatively 1000 words. Cancer patients (CP) and the simulator utterances include about 2500 words. A statistically significant decrease overtime of the mean total word used by HCP is noticeable in simulated interviews (SI) (time effect Manova *F* value: 13.96; *P*<0.001). Simultaneously, a statistically significant increase of word used by the simulator has been found (time effect Manova *F* value: 14.08; *P*=0.001). No group by time effects are noticed.

In CI, ‘anxiety’ Martindale Regressive Imagery Dictionary (MRID) subcategory and ‘distress’ Harvard Third Psychosociological Dictionary (HPSD) subcategory frequency scores are positively correlated with emotional depth level (EDL) of utterances assessed by the Cancer Research Campaign Workshop Evaluation Manual (CRCWEM) (respectively *r*=0.40 (*P*=0.000) and *r*=0.29 (*P*=0.003)). ‘Positive affect’ MRID subcategory frequency scores are negatively correlated with the EDL scores (*r*=−0.28; *P*=0.005). No significant correlations between dictionaries scores and EDL scores are noticeable in SI.

HCP which have been attending TW use more emotional words (frequency scores) tagged by the ‘distress’ HPSD subcategory, compared to untrained HCP, in CI and SI (group by time effect Manova *F* value: 2.93 (*P*=0.056) and 8.82 (*P*=0.000), respectively for CI and SI). In SI, trained HCP use different emotional words (density scores) tagged by the ‘distress’ HPSD subcategory compared to untrained HCP (group time effect Manova *F* value: 3.30 (*P*=0.041)). In CI, trained HCP use different words (density scores) tagged by ‘anxiety’ MRID subcategory compared to untrained HCP (group by time effect Manova *F* value: 3.66 (*P*=0.028)). (Table 1

**Table 1:**
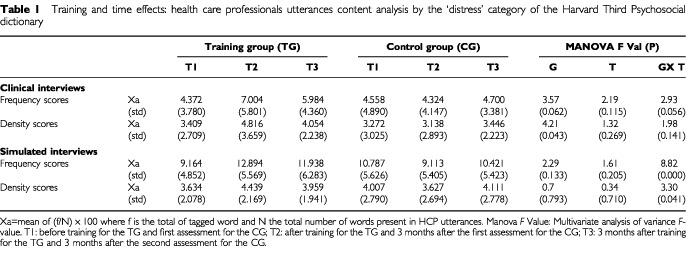
Training and time effects: health care professionals utterances content analysis by the ‘distress’ category of the Harvard Third Psychosocial dictionary

).

CP who have been communicating with trained HCP use more emotional words (frequency scores) tagged by the ‘distress’ HPSD subcategory compared to CP who have been communicating with untrained HCP (group by time effect Manova *F* value: 5.42 (*P*=0.005)). CP who have been communicating with trained HCP also use more different emotional words (density scores) tagged by the ‘distress’ HPSD subcategory compared to CP who have been communicating with untrained HCP (group by time effect Manova *F* value: 5.93 (*P*=0.003)). (Table 2

**Table 2:**
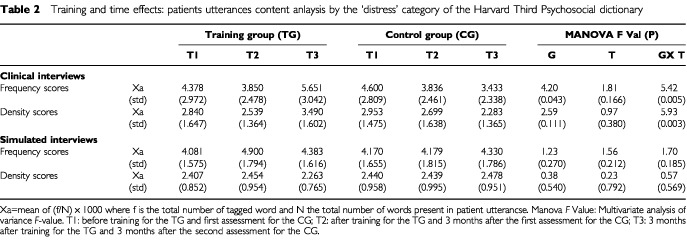
Training and time effects: patients utterances content anlaysis by the ‘distress’ category of the Harvard Third Psychosocial dictionary

).

**Figure 1:**
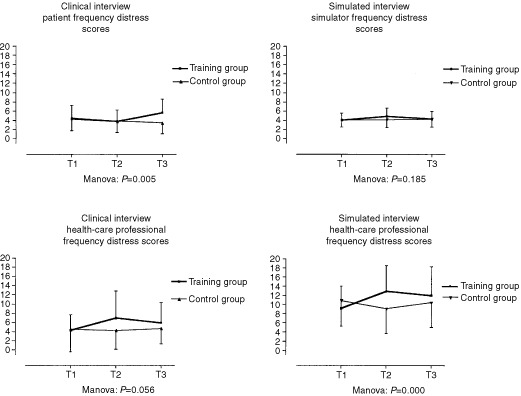
Evolution over time in control and training groups of simulator, patients and health-care professionals distress frequency scores in clinical and simulated interviews.

The simulator uses significantly less different words (density scores) tagged with the ‘aggression’ MRID subcategory in interviews with trained HCP *vs* the interviews with untrained HCP (group by time effect Manova *F* value: 3.40, *P*=0.035). Figure 1

summarizes the evolution over time of simulator, patients and health care professionals distress frequency scores in clinical and simulated interviews.

### Correlations

In CI, for ‘distress’ subcategory of the HPSD scores, correlations between trained HCP and their CP are higher especially right after the end of TW (T2) (*r*=0.75; *P*=0.000) and 3 months after the end of TW (T3) (*r*=0.39; *P*=0.005**)** than are correlations between untrained HCP and with their CP (T2) (*r*=0.25; *P*=0.068) and (T3) (*r*=0.31; *P*=0.023). In SI, these correlations are also higher for trained HCP 3 months after the end of TW (T3) (*r*=0.41; *P*=0.002) *vs* the correlations between scores of untrained HCP with their CP (T3) (*r*=0.18; *P*=0.204). These correlations are similar for trained HCP right after the end of TW (T2) (*r*=0.12; *P*=0.377) *vs* the correlations between scores of untrained HCP with their CP (T2) (*r*=0.12; *P*=0.382) (Figure 1).

In CI, for ‘anxiety’ subcategory of the MRID scores correlations between trained HCP and their CP are higher especially right after the end of TW (T2) (*r*=0.58; *P*=0.000) than the correlations between untrained HCP and their CP (T2) (*r*=0.15; *P*=0.279). These correlations are similar for trained HCP 3 months after TW (T3) (*r*=0.42; *P*=0.002) *vs* the correlations between scores of untrained HCP and their CP (T3) (*r*=0.42; *P*=0.001). In SI, these correlations are higher for trained HCP 3 months after the end of TW (T3) (*r*=0.29; *P*=0.032) than the correlations between scores of untrained HCP and their CP (T3) (*r*=0.10; *P*=0.473). These correlations are similar for trained HCP right after the end of TW (T2) (*r*=0.33; *P*=0.012) *vs* the correlations between scores of untrained HCP and their CP (T2) (*r*=0.32; *P*=0.006).

In CI, for ‘aggression’ subcategory of the MRID scores, correlations between trained HCP and their CP are similar, especially right after the end of TW (T2) (*r*=0.45; *P*=0.000) as the correlations between untrained HCP and their CP (T2) (*r*=0.46; *P*=0.000). These correlations are lower for trained HCP 3 months after TW (T3) (*r*=0.25; *P*=0.074), compared to correlations between scores of untrained HCP and their CP (T3) (*r*=0.56; *P*=0.000). In SI, correlations of ‘aggression’ MRID subcategory scores between trained HCP and simulators scores are lower right after the end of TW (T2) (*r*=0.21; *P*=0.128) and 3 months after TW (T3) (*r*=0.10; *P*=0.477) than correlations between untrained HCP scores and simulator scores (T2) (*r*=0.30; *P*=0.023) and (T3) (*r*=0.34; *P*=0.016).

In CI, for ‘sadness’ subcategory of the MRID scores, correlations between trained HCP and their CP are higher, especially right after the end of TW (T2) (*r*=0.67; *P*=0.000), than the correlations between untrained HCP and their CP (T2) (*r*=0.03; *P*=0.827). These correlations are similar for trained HCP 3 months after TW (T3) (*r*=0.20; *P*=0.162) as the correlations between scores of untrained HCP and their CP (T3) (*r*=0.27; *P*=0.046). In SI, these correlations are lower for trained HCP right after the end of TW (T2) (*r*=0.06; *P*=0.686), than correlations between scores of untrained HCP and their CP (T2) (*r*=0.39; *P*=0.003). Three months after TW these correlations become higher for trained HCP (T3) (*r*=0.26; *P*=0.065) than the correlations between scores of untrained HCP and their CP (T3) (*r*=0.15; *P*=0.295).

In CI, for ‘positive affect’ subcategory of the MRID scores, correlations between trained HCP and their CP are lower right after the end of TW (T2) (*r*=0.37; *P*=0.006) and 3 months after the end of TW (T3) (*r*=0.47; *P*=0.001) than the correlations between untrained HCP and their CP (T2) (*r*=0.51; *P*=0.000) and (T3) (*r*=0.58; *P*=0.000). In SI, these correlations are higher for trained HCP right after the end of TW (T2) (*r*=0.37; *P*=0.007) than the correlations between scores of untrained HCP with their CP (T2) (*r*=0.00; *P*=0.993). These correlations become lower, for trained HCP 3 months after the end of TW (T3) (*r*=0.10; *P*=0.466) than the correlations between scores of untrained HCP with their CP (T3) (*r*=0.59; *P*=0.000).

## DISCUSSION AND CONCLUSION

Health care professionals (HCP) frequently block cancer patients (CP) expression of emotions instead of facilitating emotional distress/concerns expressions. There is a known reciproqual influence between HCP (implicit or explicit) clarification of CP emotions and CP (implicit or explicit) expression of emotions. Marginally, it may be observed in the process reported here, an expression of HCP emotions and a clarification of HCP emotions by cancer patients.

Counting emotional words with ‘distress’, ‘anxiety’, ‘sadness’, ‘aggression’, ‘positive affect’ dictionaries and assessing the emotional depth level (EDL) of utterances with the CRCWEM allow to better understand HCP communication in general and the blocking processes in particular, which inhibit CP emotional distress/concerns expressions. To assess changes induced by TW, emotional word frequencies in SI and CI were counted before training, right after training and 3 months after training for the trained subjects on the one hand, and on the other hand, control subjects were assessed shortly after the study entry, 3 months after the first assessment, and 3 months after the second assessment. Numbers of tagged words in HCP, CP and simulator utterances were also correlated. The results of this study confirms the poor emotional content of communication. At baseline HCP use about 4.3 (std : 3.7) emotional words per thousand words used and only 3.4 (std : 2.7) different emotional words (HCP utterances of a 20 min interview includes about 1000 words). This poor loading of emotional words in HCP utterances indicates not only a limited use of emotional words but also a low access to the emotional word registry during interviews. The results also show that there is an increased use of emotional words by HCP right after having been trained : subjects in the TW arm use 4.3 (std : 3.7) emotional words per 1000 used before TW, and 7.0 (std : 5.8) right after TW and 5.9 (std : 4.3) 3 months later compared to subjects in the CG which uses 4.5 (std : 4.8) emotional words at the first assessment point, 4.3 (std : 4.1) at the second one and 4.4 (std : 3.3) at the third one (*P*=0.056). This increased use of emotional words by trained HCP facilitates CP emotional words expression. Cancer patients interacting with trained HCP use 4.3 (std : 2.9) emotional words per 1000 used before TW, 3.85 (std : 2.5) right after TW, and 5.6 (std : 3.0) 3 months later compared to cancer patients interacting with untrained HCP which use 4.6 (std : 2.8) emotional words at the first assessment point, 3.8 (std : 2.4) at the second one and 3.4 (std : 2.3) at the third one. The same trend is noticeable for emotional words used by HCP in simulated interviews (*P*=0.000). The emotional words registry used by HCP remains however stable over time in clinical interviews (*P*=0.141) and is enlarged in simulated interviews (*P*=0.041). Finally, trained HCP use less neutral utterances than the untrained HCP (*P*=0.055). The ‘distress’ category of the HPSD appears thus to be the most sensitive to post-TW changes, compared to emotional subcategories of MRID and to the CRCWEM rating of ‘emotional’ depth level. It is important to underline at this level that there were no differences in the EORTC QLQ-C30 emotional functioning scores of cancer patients which participated to the three assessment points.

Correlation of dictionary scores with the ‘emotional’ depth level (EDL) of utterances scores (assessed by the CRCWEM) specifies the respective benefits of the two scoring methods. For CI, as expected, significant correlations were observed: ‘positive affect’ subcategory of the MRID dictionary scores are negatively correlated with the EDL scores and ‘anxiety’ subcategory of the MRID and ‘distress’ subcategory of the HPSD scores are positively correlated with the EDL scores. However the CRCWEM rating of EDL of utterances is not highly correlated with the emotional tone of interviews assessed by dictionary subcategory scores. There are some reasons which may explain these findings. Firstly, computer-supported content analysis with dictionaries assesses words and not meanings: ‘I am depressed’ or ‘I am not depressed’ generates the same score with a computer-supported content analysis but not with the CRCWEM rating system. Secondly and moreover the CRCWEM rating system assesses emotions globally. Meanwhile, this rating system allows to assess implicit emotions included in utterances even if emotional words are not expressed.

The results show on one hand that trained HCP and on the other hand CP interacting with trained HCP, use more ‘emotional’ words compared to untrained HCP and CP interacting with untrained HCP. The ‘distress’ subcategory of the HPSD seems to be a useful dictionary subcategory, able to tag words likely to be sensitive to the effect of TW. The ‘distress’ subcategory has also allowed to assess the frequency of ‘distress’ words used by HCP right after TW and 3 months later : trained HCP use more words tagged by the ‘distress’ subcategory of the HPSD.

Compared to other dictionary subcategories, the ‘distress’ subcategory of HPSD seems to be the most sensitive tool for assessing TW aiming at improving CS. Its higher sensitivity to TW induced changes may be due to the following reasons. Firstly, it should be recalled that the ‘distress’ subcategory of the HPSD contains 210 words. This subcategory is therefore more likely to tag emotional words compared to other MRID subcategories which respectively contain 46 (‘anxiety’), 55 (‘sadness’) and 73 (‘positive affect’) words. Secondly, ‘distress’ subcategory certainly tags emotional words that are the most frequently used in a clinical context, whereas the ‘aggression’ subcategory of MRID which contains about the same number of words (211 words) is less likely to tag words commonly used in clinical contexts.

Dictionaries which produce scores for defined emotional subcategories may discriminate between specific emotional feelings such as anxiety, sadness, aggression and distress. It is obvious that it would be difficult and time-consuming to obtain a high interrater reliability for raters assessing these specific emotional subcategories. The ‘aggression’ MRID subcategory may be useful for example in some clinical contexts (expression of anxiety or depression with irritation or anger words). In this study in simulated interviews, the simulator has expressed, to a certain extent, her anxiety by using ‘anger’ words which have been tagged by the ‘aggression’ MRID subcategory. Counting the number of ‘aggression’ words was therefore potentially useful. The results show that trained HCP react less with ‘aggression’ words to the simulator ‘anger’ expression than do untrained HCP.

The HPSD ‘distress’ subcategory seems to be a good tool to assess HCP empathy before and after CS-TW. It is useful to recall that the ‘distress’ subcategory of HPSD contains ‘sadness’, ‘anxiety’, and ‘aggression’ words which may be expressed by patients when they experience emotional distress/concerns. To assess further more experienced or already trained HCP communication skills, the use of ‘positive affect’, ‘anxiety’, ‘sadness’ and ‘aggression’ MRID subcategory dictionaries will probably give an insight on more specific HCP empathy abilities for certain types of concerns/distress emotion and in certain type of clinical contexts.

The calculation of density scores assesses HCP and CP emotional lexical registries. In the present study, following TW there is an increased frequency of emotional words used by HCP and an increased frequency and density of emotional words used by CP. Thus an increased frequency of emotional words used by HCP seems to facilitate CP expression of emotions not only quantitatively (higher number of emotional words expressed; frequency scores) but also qualitatively (higher number of different emotional words expressed; density scores). The fact that the density of emotional words used by HCP in SI increases after TW may indicate that HCP feel more comfortable in using a larger emotional lexical registry. It may be hypothesised that TW targetting more precisely on this issue will allow the transfer to CI of this type of skill.

Is it possible to enhance communication skills in general and empathy in particular? This study has assessed prospectively the effect of TW on HCP and CP expression of emotions. The professional empathy process is clearly enhanced by TW, at least a part of it : particularly skills which need the use of emotional words such as emotions recognition, clarification, discrimination. This study also highlights the further work needed to amplify the efficacy of TW. In particular, it may be suggested to design TW targetted to enlarge HCP emotional words registry in order to achieve this increased efficacy. Counting words with dictionaries has allowed to assess quantitatively the benefit of a TW at the level of HCP facilitation of CP expression of emotions.

Dictionaries also allow to assess the emotional tone of simulator performances. In this study, the simulator have stable ‘distress’ scores. This stability reflects the actress standardisation of her role.
